# Beta-Blockers in Patients with Myocardial Infarction and Preserved Left Ventricular Ejection: A Systematic Review and Meta-Analysis of Randomized Controlled Trials

**DOI:** 10.3390/jcm14010150

**Published:** 2024-12-30

**Authors:** Michael Sabina, Shrinand Shah, Mason Grimm, Jean Carlo Daher, Paola Campillo, Mohammed Baraa Boozo, Ahmad Al-Abdouh, Waiel Abusnina, Fabrizio D’ Ascenzo, Anas Bizanti

**Affiliations:** 1 Lakeland Regional Health Medical Center, Lakeland, FL 33805, USA; shrinand.shah@mylrh.org (S.S.); mason.grimm@mylrh.org (M.G.); jean.carlodaher@mylrh.org (J.C.D.); paola.campillo@mylrh.org (P.C.); anas.bizanti@mylrh.org (A.B.); 2 Department of Metabolism and Physiology, H. Lee Moffitt Cancer Center & Research Institute, Tamp, FL 33612, USA; mohammadbaraa.boozo@moffitt.org; 3 Departments of Medicine, University of Kentucky, Lexington, KY 40536, USA; ahmad_alabouh@yahoo.com; 4 Medstar Georgetown University Hospital, Washington, DC 20007, USA; waiel.abusnina@gmail.com; 5 Division of Cardiology, Cardiovascular and Thoracic Department, AOU Città della Salute e della Scienza di Torino and University of Turin, 10126 Turin, Italy; fabrizio.dascenzo@gmail.com

**Keywords:** beta-blockers, myocardial infarction, preserved left ventricular ejection fraction, percutaneous coronary intervention, systematic review and meta-analysis

## Abstract

**Background:** The benefit of beta-blockers (BBs) for myocardial infarction (MI) patients with a preserved left ventricular ejection fraction (LVEF) is uncertain. While beneficial for a reduced LVEF, their efficacy with a preserved LVEF, especially with modern revascularization, is unclear. **Methods:** A PRISMA-guided systematic review and meta-analysis utilized PubMed and EMBASE. Three randomized controlled trials comparing outcomes in MI patients with a preserved LVEF treated with BBs versus no treatment were included. The primary outcome was composite all-cause mortality and MI; secondary outcomes were all-cause mortality, cardiovascular mortality, MI, and stroke. **Results:** Three studies, including a total of 9512 participants, were analyzed. Beta-blockers did not demonstrate a statistically significant benefit in reducing the composite endpoint of all-cause mortality and myocardial infarction (RR 0.97, 95% CI: 0.84–1.12, *p* = 0.671, I^2^ = 0%). Similarly, no significant effect was observed for secondary outcomes: all-cause mortality (RR 0.96, 95% CI: 0.79–1.17, *p* = 0.708), cardiovascular mortality (RR 1.22, 95% CI: 0.87–1.72, *p* = 0.247), myocardial infarction (RR 0.97, 95% CI: 0.78–1.19, *p* = 0.759), and stroke (RR 0.96, 95% CI: 0.66–1.38, *p* = 0.819). **Conclusions:** In patients with myocardial infarction and a preserved LVEF, beta-blockers did not significantly reduce mortality, recurrent myocardial infarction, or stroke, suggesting a limited benefit in this population under contemporary management protocols.

## 1. Introduction

The clinical benefit of beta-blockers after a myocardial infarction (MI) in patients with a reduced LVEF is well-established [1,2,3]. Beta-blockers have long been a cornerstone of secondary prevention following acute MI, with landmark studies in the 1980s demonstrating a significant reduction in mortality [4,5,6]. These benefits are largely attributed to beta-blockers’ negative chronotropic and inotropic effects, which reduce the oxygen demand on cardiac myocytes [7,8]. However, with advances in revascularization and improved medical therapy for secondary prevention, the prognosis of MI patients has improved, leading to questions about the necessity of long-term beta-blocker therapy in patients without a reduced LVEF and heart failure [9,10]. The introduction of primary PCI has resulted in increased salvage of the myocardium, creating a smaller ischemic substrate and a myocardium less susceptible to arrhythmias [11,12]. Consequently, the role of beta-blockers in contemporary post-MI patients without heart failure has come under scrutiny [13,14]. Early RCTs conducted in the 1980s and 1990s documented a significant reduction in mortality with beta-blocker therapy post-MI [15]. However, these trials were conducted before revascularization became standard practice, and more recent meta-analyses have shown that the mortality benefit of beta-blockers is primarily evident in the pre-reperfusion era [16]. In the reperfusion era, only one RCT (CAPITAL-RCT) has specifically investigated the long-term efficacy of beta-blockers in patients with an LVEF ≥ 40% after MI and successful PCI, finding no significant benefit [14]. Given the lack of contemporary RCTs addressing long-term beta-blocker therapy in MI patients without heart failure, observational and registry studies have been summarized in meta-analyses, yielding mixed results [17,18,19,20,21,22]. For example, while some studies suggest a mortality benefit associated with long-term beta-blocker use, this benefit often disappears when controlling for biases, highlighting the need for more rigorous evidence [20]. The absence of consistent data and the evolution of MI management have led to differing recommendations in contemporary guidelines. The 2021 ACC/AHA guidelines rate beta-blockers as a class III recommendation post-revascularization [23]. The European Society of Cardiology (ESC) recommends beta-blockers for in-hospital use and continuation post-discharge in patients without contraindications, though the level of evidence varies [18,24]. In contrast, the NICE guidelines recommend beta-blocker therapy for at least one year after MI in patients without a reduced LVEF or heart failure [25]. While beta-blockers are generally well-tolerated, they are associated with common side effects that may negatively impact quality of life [26,27]. Lower compliance with beta-blocker treatment compared to other cardiovascular medications may be partly due to these side effects, although not all are substantiated by evidence from randomized trials [28,29]. This study aims to critically evaluate the long-term efficacy of beta-blockers in contemporary post-MI patients with a preserved ejection fraction, addressing current gaps in evidence and informing future clinical guidelines.

## 2. Materials and Methods

### 2.1. Data Source and Searches

**Figure 1:**
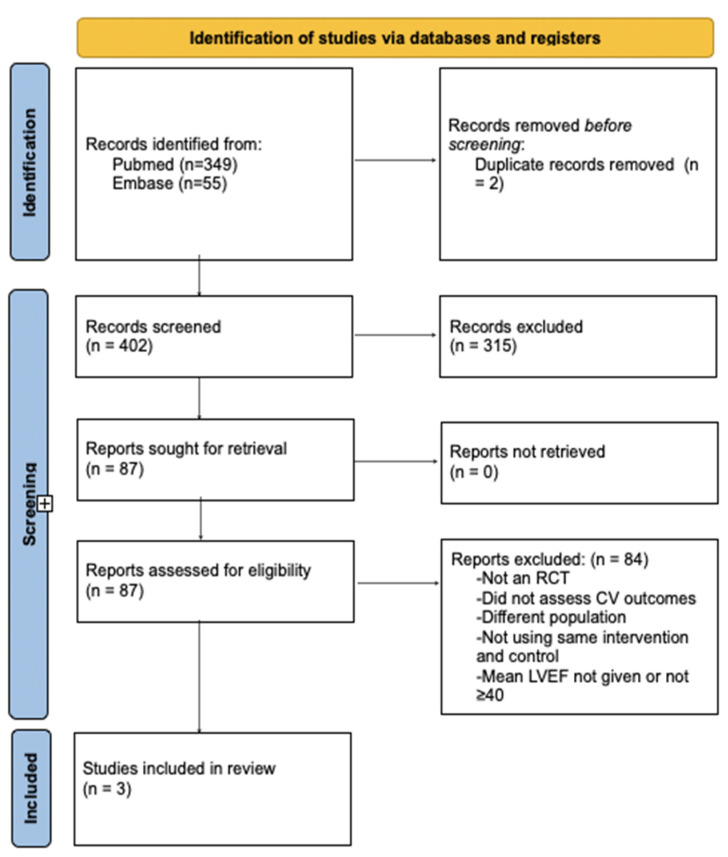
PRISMA flow diagram.

We followed the PRISMA guidelines and have summarized our search process in Figure 1. Adhering to the PRISMA checklist, we ensured the comprehensive and transparent reporting of our systematic review, as seen in the File S1 [30]. Two investigators (M.S., and M.G.) developed the search strategy, which was revised and approved by the other investigators. We searched the following databases all time until 1 August 2024: PubMed-MEDLINE and EMBASE-OVID. The search strategy is shown in the Supplementary File. There was no language limitation.

### 2.2. Study Selection

We included only RCTs in English reporting benefit or harm outcomes of long-term beta-blockers as the treatment in patients with MI and a preserved LVEF. We excluded studies that did not match our population, intervention, and outcomes. Three investigators (M.S., M.G., and S.S.) independently screened each record title and abstract for potential inclusion. They then assessed the full texts of selected abstracts. Discrepancies were resolved through discussion or by another investigator (A.B.).

### 2.3. Outcomes

Primary outcomes were death from any cause or MI. Secondary outcomes were death from any cause, death from a cardiovascular (CV) cause, MI, and stroke.

### 2.4. Data Extraction

Three investigators (M.S., M.G., and S.S.) independently extracted data on the following variables: study year, design, setting, country(ies), and sample size. Clinical characteristics included the median age (IQR), female sex, body mass index (kg), and risk factors, such as smoking, hypertension, dyslipidemia, chronic obstructive pulmonary disease (COPD), hemoglobin (g/dL), eGFR (mL/min/1.73 m^2^), hemodialysis, malignancy, and diabetes mellitus (insulin-treated). Data on previous cardiovascular diseases were recorded, including myocardial infarction, PCI, CABG, stroke, and heart failure. Presentation characteristics covered chest pain, CPR before hospital arrival, pulmonary rales, the median heart rate (IQR), the systolic blood pressure (IQR), atrial fibrillation, and STEMI (anterior, inferior/posterior, lateral). We also recorded current oral beta-blocker treatment and the median days from admission to randomization (IQR). In-hospital course variables included coronary angiography results, vessel disease, PCI, staged PCI, coronary artery bypass graft (CABG), a drug eluting stent (DES), and a bare metal stent (BMS). Finally, discharge medications were noted, including aspirin, P2Y12 receptor blockers, cilostazol, beta-blockers, ACE inhibitors or ARBs, statins, diuretics, calcium-channel blockers, aldosterone antagonists, nitrates, nicorandil, warfarin, PPIs, and H2 blockers. Discrepancies were resolved through discussion or by another investigator (A.B.).

### 2.5. Risk of Bias Assessment

Two investigators (M.S. and M.G.) independently assessed risk of bias (RoB) by using the Cochrane RoB 2.0 tool [31]; disagreements were resolved through discussion with a third investigator (S.S.). The RoB per domain and per study were described as low, moderate, serious, critical, and no information for non-randomized studies and as low, some concerns, and high for RCTs. The risk of bias assessment can be viewed in our Supplementary File.

### 2.6. Statistical Analysis

We conducted our systematic review in accordance with the 2020 PRISMA guidelines and checklist, which can be seen in our Supplementary File [30]. All meta-analyses were performed using the meta package of RevMan 5.1 [32]. We quantified heterogeneity among studies using the I^2^ statistic, with an I^2^ > 60% indicating substantial heterogeneity. To assess the robustness of our findings, we performed a leave-one-out sensitivity analysis, which can be seen in our Supplementary File. The quality of evidence was assessed using the GRADE methodology, which evaluates five key aspects: risk of bias, inconsistency, indirectness, imprecision, and publication bias [33]. Publication bias was not assessed given that we only had three studies.

## 3. Results

### 3.1. Selection of Studies

A comprehensive search was conducted on 5 August 2024, using PubMed and Embase to identify relevant citations. The search included the MeSH terms “Myocardial Infarction” and “Percutaneous Coronary Intervention” combined with “Adrenergic beta-Antagonists” to capture trials involving patients with myocardial infarction treated with beta-blockers or the standard of care. The PICO system on EMBASE was similarly used with the terms “myocardial infarction”, “percutaneous coronary intervention”, “beta-adrenergic receptor blocking agent”, and related synonyms, in combination with the placebo as a comparison and the RCT term for the study design. A total of 404 citations were identified (349 from PubMed and 55 from EMBASE). Following this, 2 duplicates were removed, leaving 402 results for additional screening. These were further filtered by type of study (RCT) leaving 87 studies to be assessed for eligibility. During the screening process, 84 studies were excluded based on criteria, such as not using the intervention, having different controls, different populations, or different endpoints (e.g., mean LVEF not given or not ≥40, or not compared to no beta-blocker as a control). Three (*n* = 3) studies were selected for the analysis. The included studies’ designs are detailed in Table 1, and the baseline characteristics of each individual study are summarized in Table 2.

### 3.2. Characteristics of Included Studies

The characteristics of the included studies are summarized in Table 1, focusing on three randomized controlled trials (RCTs). These trials were conducted in Japan, Sweden, Estonia, New Zealand, and France, with inclusion periods spanning from 2010 to 2023. The number of patients in each study varied, with substantial sample sizes in the beta-blocker (BB) and no beta-blocker (No BB) groups. The reported mean left ventricular ejection fraction (LVEF) values ranged from 58% to 60%, and follow-up periods varied between 3 to nearly 4 years. Carvedilol, Metoprolol, Bisoprolol, Acebutalol, and Atenolol were the primary beta-blockers used, with Carvedilol being exclusively used in the CAPITAL-RCT, while REDUCE-AMI and ABYSS featured varied beta-blocker distributions.

### 3.3. Baseline Characteristics

The baseline characteristics across the three studies indicate a relatively consistent median age among patients, ranging from 63 to 65 years, with a narrow interquartile range, suggesting a homogenous age distribution. Female representation was notably low, with women comprising only about 17% to 22.6% of the study populations, indicating a predominantly male cohort. Cardiovascular risk factors were prevalent, with hypertension affecting approximately 46% to 61% of patients, while the proportion of current smokers ranged from 18.5% to 47%. Notably, a significant proportion of patients had a history of prior cardiovascular interventions, with particularly high rates of previous percutaneous coronary interventions (PCI) in the REDUCE-AMI cohort (96.4% to 97.4%). At discharge, adherence to guideline-directed medical therapy was high, as evidenced by the nearly universal prescription of aspirin (94.6% to 98%) and statins (95.8% to 99%), alongside the substantial use of ACE inhibitors/ARBs, reflecting efforts to optimize secondary prevention. Overall, the populations presented in Table 2 are well-balanced, with no significant differences across these baseline characteristics.

### 3.4. Outcomes

**Figure 2:**
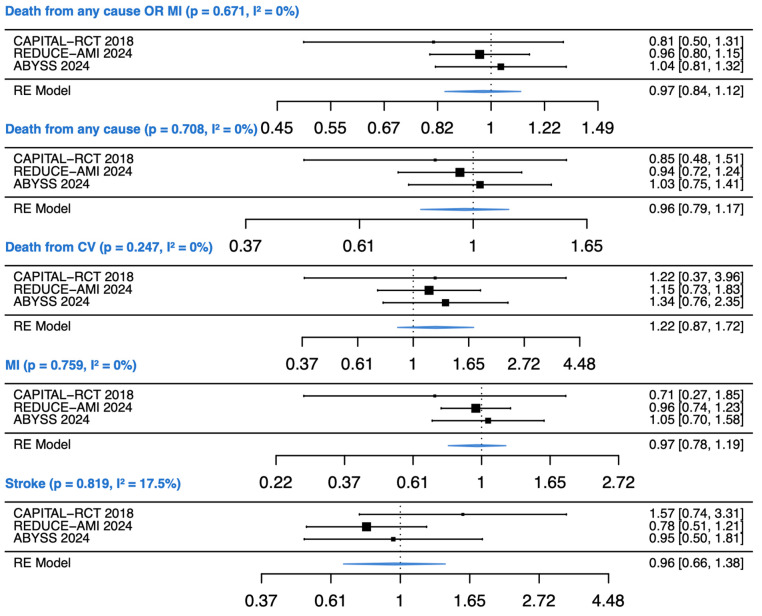
Forest plot of primary and secondary outcomes [14,34,35].

The forest plots in Figure 2 offer a detailed comparison of the effects of BB versus no BB on primary and secondary outcomes. For the primary outcome—death from any cause or myocardial infarction (MI)—the pooled estimate shows no statistically significant difference between the BB and no-BB groups, with a combined RR of 0.97 (95% CI: 0.84–1.12, *p* = 0.671, I^2^ = 0%). For all-cause mortality, the combined RR is 0.96 (95% CI: 0.79–1.17, *p* = 0.708, I^2^ = 0%), indicating no significant reduction in risk with BB use. When considering cardiovascular mortality, the RR is 1.22 (95% CI: 0.87–1.72, *p* = 0.247, I^2^ = 0%), again showing no significant effect of BB on reducing cardiovascular death. For MI as a secondary outcome, the RR is 0.97 (95% CI: 0.78–1.19, *p* = 0.759, I^2^ = 0%), indicating no significant benefit. Lastly, for stroke, the RR is 0.96 (95% CI: 0.66–1.38, *p* = 0.819, I^2^ = 17.5%), suggesting no significant reduction in stroke incidence with BBs. These findings align with the specific outcomes presented in Table 3, which details the results for each individual trial included in this analysis.

### 3.5. Risk of Bias of Included Studies

The ABYSS study shows some concerns for bias primarily due to its open-label design, which allowed for flexibility in interventions. Despite this, the study’s centralized randomization, blinded outcome adjudication, and low missing data help maintain reliability. Similarly, REDUCE-AMI presents low to some concerns, with strong randomization and minimal missing data but some performance bias from flexible dosing. In contrast, CAPITAL-RCT demonstrates a low risk of bias overall, with rigorous randomization, high adherence, and independent adjudication that support its reliability even with an open-label approach. The ABYSS study provides moderate-quality evidence, with some concerns due to its open-label design and minor imprecision in effect estimates. REDUCE-AMI and CAPITAL-RCT both provide high-quality evidence, with strong randomization, large sample sizes, and outcomes directly relevant to clinical practice. While REDUCE-AMI has a low-to-some-concerns risk of bias from its flexible dosing approach, its consistency and directness support robust findings. CAPITAL-RCT shows a low risk across all domains, with rigorous controls and precise, consistent results that strengthen the reliability of its conclusions. We did not perform an eggers test to assess publication bias given that there are only three studies. This risk of bias analysis is summarized in S5. We conducted a sensitivity analysis to evaluate the impact of BB therapy on primary and secondary outcomes (S4). This included a leave-one-out analysis for each trial. The leave-one-out sensitivity analysis shows that removing any single study (CAPITAL-RCT, REDUCE-AMI, or ABYSS) does not significantly change the pooled results for outcomes like death from any cause and MI, all-cause death, CV death, MI, or stroke, indicating robustness and stability of the overall findings.

## 4. Discussion

This meta-analysis incorporated data from three RCTs, including a total of 9512 participants. We found that beta-blockers were not effective in reducing the composite endpoint of all-cause mortality and MI, all-cause mortality, cardiovascular mortality, reinfarction, and stroke compared to no beta-blocker therapy in patients who presented with MI and a preserved ejection fraction at the time of revascularization.

### Limitations

Although the aim of this study was to comment on the use of beta-blockers in MI patients with a preserved left ventricular function who had undergone PCI, it is essential to note the disparities in inclusion criteria for studies in our analysis. REDUCE-AMI is the only trial that excluded patients with ejection fractions below 50%. Supplementary data on ABYSS revealed that the study included patients within the mildly reduced ejection fraction subgroup (40–50%), 170 of 1812 within the beta-blocker interruption arm, and 168 of 1821 within the beta-blocker continuation arm. However, despite this, only the stroke outcome experienced heterogeneity when data from all three trials were pooled. The CAPITAL-RCT trial did not have supplementary data to show ejection fraction subgroups. Still, their inclusion criteria included patients with an ejection fraction greater than 40%, which includes both HFmrEF and HFpEF. Unfortunately, we are unable to quantify sample sizes for each subgroup.

In stable ischemic heart disease with a normal left ventricular function, beta-blockers are currently a class IIIa recommendation, per the ACC/AHA, in patients who have undergone revascularization, indicating that they are thought to have no benefit [23]. Given the data extrapolated in this study, it is now vital to consider the harm of prescribing beta-blockers. The REDUCE-AMI trial examined safety endpoints among its patients, comparing hospitalization rates for conditions, such as bradycardia, a second or third-degree AV block, hypotension, syncope, pacemaker implantation, and hospitalization for asthma or COPD [34]. No significant difference in the rate of occurrence was found; however, it is important to note that this trial was not powered to scrutinize this topic either. Neither the CAPITAL-RCT trial nor the ABYSS trial included an assessment of adverse events within the outcomes [14,36]. Future RCTs, including REBOOT-CNIC, BETAMI, and DANBLOCK, should examine safety endpoints [36,37,38], allowing for a robust analysis of beta-blocker safety and harms, not only the benefit or lack thereof. These studies will assess unique safety endpoints that will add new data to the literature.

The current recommendations indicating that beta-blockers are of no benefit for patients who have undergone PCI in the 2021 ACC/AHA guidelines are supported by observational studies on the National Cardiovascular Data Registry (NCDR) published in 2016 and on the REACH registry published in 2012 [39,40]. Both studies are inherently inferior data points to the randomized control trials that are now available to support guideline recommendations given the inability to control for factors that may influence the data. Of note the NCDR study excluded patients with ejection fractions less than 40%, resulting in the inclusion of patients in the mildly reduced category, but it has been used as evidence for guiding therapies for patients with a preserved function. Additionally, the authors disclose the inability to track drug claim data and adjust for possible biases, such as a lack of compliance, the use of suboptimal dosing, types of beta-blockers, and changes in the pattern of use over time. In the 2012 study on the REACH registry, the authors note that a history of heart failure was controlled for; however, they did not record or stratify patients based on the ejection fraction [40]. Again, a study with no data on specific classifications of left ventricular function is being used for evidence-based commentary on patients with a preserved function. The limitations in both studies highlight the need for new trials to provide data that can be used to update the guidelines.

Three new randomized control trials were identified that will attempt to gather further data on this topic. All three studies will include patients with an LVEF greater than 40%; however, data will also be stratified based on ejection fractions of 40–49% and greater than 50%. These data can be used to update guidelines on patients post-MI with a preserved left ventricular function and begin developing recommendations for those with a mild reduced function. Additionally, BETAMI and DANBLOCK will assess the unique adverse effects of beta-blockers.

Another significant limitation of our analysis is the sex disparity in the study populations, with women comprising only 17–23% of participants. This underrepresentation limits the generalizability of our findings to female patients, who may exhibit different responses to beta-blocker therapy.

## 5. Conclusions

In patients with MI and a preserved ejection fraction who underwent PCI, beta-blockers did not show a reduction in mortality, recurrent MI, or stroke. Despite this lack of benefit, beta-blockers are still widely prescribed for all MI patients, even when guidelines acknowledge that there is no advantage for those with a preserved LV function. Future research should prioritize evaluating safety outcomes to determine whether this broad prescription approach may actually be causing harm.

## Figures and Tables

**Table 1:** Study characteristics.

Study	Design	Country	Inclusion Period	Number of Patients (BB/No BB)	Mean LVEF (%)	Follow-Up Time	Beta-Blocker Used
CAPITAL-RCT 2018	RCT	Japan	2010–2014	394/400	58	3.9 * years	Carvedilol (100%)
REDUCE-AMI 2024	RCT	Sweden (95.4%), Estonia, New Zealand	2017–2023	2508/2512	NA	3.5 * years (2.2–4.7)	Metoprolol (62.2%) and Bisoprolol (37.8%)
ABYSS 2024	RCT	France	2018–2022	1772/1656	60 IQR (52–60) *	3.0 years IQR (2–4) *	Bisoprolol (71.5%), Acebutalol (10.7%), and Atenolol (9.1%)

* Given as the median. RCT = randomized controlled trial; IQR = interquartile range.

**Table 2:** Baseline characteristics.

Study	CAPITAL-RCT 2018		REDUCE-AMI 2024		ABYSS 2024	
Sample Size	BB (*N* = 394)	No BB (*N* = 400)	BB (*N* = 2508)	No BB (*N* = 2512)	BB (*N* = 1852)	No BB (*N* = 1846)
Median age (IQR)—yr	63.9 (51.7–75.1)	64.5 (53.2–75.8)	65 (57–73)	65 (57–73)	63.5 ± 10.9	63.5 ± 11.2
Female sex—no. (%)	67 (17)	88 (22)	563 (22)	568 (23)	316 (17)	307 (17)
Body mass index (kg)	24.0 ± 3.3	23.9 ± 3.2	–	–	26.5 (24.1–29.6)	26.3 (23.9–29.4)
Risk factors—no./total no. (%)					-	-
Current smoking	186 (47)	188 (47)	478/2466 (19.4)	530/2483 (21.3)	342 (18.5)	385 (20.9)
Hypertension	239 (61)	234 (59)	1155/2507 (46.1)	1163/2509 (46.4)	805 (43.5)	786 (42.6)
Dyslipidemia	191 (48)	211 (53)	-	-	994 (53.7)	948 (51.4)
COPD	5 (1.3)	7 (1.8)	-	-	-	-
Hemoglobin (g/dL)	13.2	13	-	-	-	-
eGFR (mL/min/1.73 m^2^)	-	-	-	-	-	-
Hemodialysis	2 (0.5)	1 (0.3)	-	-	-	-
Malignancy	29 (7.4)	23 (5.8)	-	-	-	-
Diabetes mellitus	98 (25)	81 (20)	346/2506 (13.8)	354/2509 (14.1)	375 (20.2)	372 (20.2)
Treated with insulin therapy	6 (1.5)	12 (3.0)	-	-	-	-
Previous cardiovascular disease—no./total no. (%)					-	-
Previous myocardial infarction	16 (4.1)	9 (2.3)	165/2503 (6.6)	192/2507 (7.7)	-	-
Previous PCI	20 (5.1)	21 (5.3)	147/2504 (5.9)	175/2505 (7.0)	1693/1757 (96.4)	1709/1755 (97.4)
Previous CABG	-	-	33/2504 (1.3)	36/2507 (1.4)	83/1757 (4.7)	62/1755 (3.5)
Previous stroke	18 (4.6)	19 (4.8)	52/2506 (2.1)	67/2507 (2.7)	67 (3.6)	56 (3.0)
Previous heart failure	-	-	13/2486 (0.5)	22/2481 (0.9)	26 (1.4)	34 (1.8)
Characteristic at presentation					-	-
Chest pain as main symptom—no./total no. (%)	-	-	2421/2507 (96.6)	2417/2512 (96.2)	-	-
CPR before hospital arrival—no./total no. (%)	-	-	10/2483 (0.4)	11/2485 (0.4)	-	-
Pulmonary rales—no./total no. (%)	-	-	29/2445 (1.2)	42/2462 (1.7)	-	-
Median heart rate (IQR)—beats/min	74.8 ± 14.3	73.2 ± 13.9	74 (65–85)	73 (64–84)	-	-
Median systolic blood pressure (IQR)—mm Hg	121 ± 21	121 ± 20	150 (135–170)	151 (136–170)	-	-
Atrial fibrillation—no./total no. (%)	-	-	21/2502 (0.8)	23/2504 (0.9)	-	-
ST-segment elevation myocardial infarction—no./total no. (%)	-	-	877/2507 (35.0)	892/2512 (35.5)	-	-
Anterior STEMI—no./total no. (%)	157 (40)	153 (38)			-	-
Inferior/posterior—no./total no. (%)	203 (52)	209 (52)			-	-
Lateral—no./total no. (%)	34 (8.6)	38 (9.5)			-	-
Current oral beta-blocker treatment—no./total no. (%)	-	-	269/2468 (10.9)	302/2472 (12.2)	-	-
Median no. of days from hospital admission to randomization (IQR)	-	-	2 (1–3)	2 (1–3)	-	-
In-hospital course—no./total no. (%)					-	-
Coronary angiography	-	-	-	-	-	-
No stenosis	-	-	26/2484 (1.0)	25/2491 (1.0)	-	-
LAD	164 (42)	172 (43)	-	-	-	-
RCA	185 (47)	185 (46)	-	-	-	-
LCX	45 (11)	43 (11)	-	-	-	-
One-vessel disease	259 (66)	264 (66)	1378/2484 (55.5)	1378/2491 (55.3)	-	-
Two-vessel disease	102 (26)	90 (23)	676/2484 (27.2)	668/2491 (26.8)	-	-
Left main or three-vessel disease	33 (8.4)	46 (12)	404/2484 (16.3)	420/2491 (16.9)	-	-
PCI	-	-	2387/2491 (95.8)	2376/2496 (95.2)	-	-
Staged PCI for non-infarct-related artery	73/393 (20)	89/399 (22)	-	-	-	-
CABG	-	-	92/2491 (3.7)	103/2496 (4.1)	-	-
DES	176/376 (43)	174/382 (46)	-	-	-	-
BMS	203/376 (54)	212/382 (56)	-	-	-	-
Medication at discharge—no./total no. (%)			-	-	-	-
Aspirin	388 (98)	393 (98)	2450/2507 (97.7)	2440/2512 (97.1)	1756 (94.8)	1746 (94.6)
P2Y12 receptor blocker	370 (94)	367 (92)	2411/2507 (96.2)	2398/2512 (95.5)	Clopidegrel 377 (20.3), Ticragelor or prasugrel 430 (23.2)	Clopidegrel 337 (18.3), Ticragelor or prasugrel 412 (22.3)
Cilostazol	5 (1.3)	6 (1.5)	-	-	-	-
Beta-blocker	-	-	2399/2505 (95.8)	247/2512 (9.8)	-	-
ACE inhibitor or ARB	296 (75)	316 (79)	1985/2507 (79.2)	2040/2512 (81.2)	1363 (73.6)	1363 (73.6)
Statin	340 (86)	345 (86)	2481/2507 (99.0)	2461/2510 (98.0)	1775 (95.8)	1775 (95.8)
Diuretic agent	-	-	211/2507 (8.4)	191/2512 (7.6)	180 (9.7)	180 (9.7)
Calcium-channel blocker	54 (14)	49 (12)	416/2508 (16.6)	496/2511 (19.8)	206 (11.1)	206 (11.1)
Aldosterone antagonist	38 (9.6)	35 (8.8)	-	-	41 (2.2)	41 (2.2)
Nitrate	44 (11)	43 (11)	-	-	-	-
Nicorandil	63 (16)	70 (18)	-	-	-	-
Warfarin	13 (3.3)	9 (2.3)	-	-	-	-
PPI	303 (77)	314 (79)	-	-	-	-
H2 blocker	51 (13)	50 (13)	-	-	-	-

BB = beta-blockers; No BB = no beta-blockers; IQR = interquartile range; yr = year; COPD = chronic obstructive pulmonary disease; eGFR = estimated glomerular filtration rate; PCI = percutaneous coronary intervention; CABG = coronary artery bypass graft; CPR = cardiopulmonary resuscitation; LAD = left anterior descending artery; RCA = right coronary artery; LCX = left circumflex artery; STEMI = ST-segment elevation myocardial infarction; ACE = angiotensin-converting enzyme; ARB = angiotensin II receptor blocker; DES = drug-eluting stent; BMS = bare-metal stent; PPI = proton pump inhibitor; H2 blocker = histamine H2 receptor antagonist.

**Table 3:** Primary and secondary outcomes.

Study	Sample Size per Arm	Death from Any Cause OR MI—No. (%)	Death from Any Cause—No. (%)	Death from CV Cause—No. (%)	MI—No. (%)	Stroke—No. (%)
CAPITAL-RCT 2018 [14]	BB (N = 394)	27	20 (3.6)	6 (1.1)	7 (2.0)	17 (4.0)
	No BB (N = 400)	34	24 (3.8)	5 (1.4)	10 (1.9)	11 (2.1)
REDUCE-AMI 2024 [34]	BB (N = 2508)	199 (7.9)	97 (3.9)	38 (1.5)	112 (4.5)	36 (1.4)
	No BB (N = 2512)	208 (8.3)	103 (4.1)	33 (1.3)	117 (4.7)	46 (1.8)
ABYSS 2024 [35]	BB (N = 1846)	122 (6.6)	76 (4.1)	28 (1.5)	46 (2.5)	18 (1.0)
	No BB (N = 1852)	118 (6.4)	74 (4.0)	21 (1.1)	44 (2.4)	19 (1.0)
Pooled result—RR (95% CI; *p*)	BB (N = 4748)	0.97 (0.84–1.12; *p* = 0.671)	0.96 (0.79–1.17; *p* = 0.708)	1.22 (0.87–1.72; *p* = 0.247)	0.97 (0.78–1.19; *p* = 0.759)	0.96 (0.66–1.38; *p* = 0.819)
	No BB (N = 4764)					

BB = beta-blockers; No BB = no beta-blockers; MI = myocardial infarction; CV = cardiovascular; RR = risk ratio; CI = confidence interval.

## Data Availability

No new data were generated or analyzed in support of this research.

## Supplementary Materials

The following supporting information can be downloaded at: https://www.mdpi.com/article/10.3390/jcm14010150/s1.
